# RecQL4 Helicase Amplification Is Involved in Human Breast Tumorigenesis

**DOI:** 10.1371/journal.pone.0069600

**Published:** 2013-07-22

**Authors:** Hongbo Fang, Linghu Nie, Zhenfen Chi, Jing Liu, Dan Guo, Xuemei Lu, Tom K. Hei, Adayabalam S. Balajee, Yongliang Zhao

**Affiliations:** 1 Laboratory of Disease Genomics and Individualized Medicine, Beijing Institute of Genomics, Chinese Academy of Sciences, Beijing, China; 2 University of Chinese Academy of Sciences, Beijing, China; 3 Department of Radiation Oncology, Center for Radiological Research, Columbia University Medical Center, New York, New York, United States of America; University of South Alabama, United States of America

## Abstract

Breast cancer occur both in hereditary and sporadic forms, and the later one comprises an overwhelming majority of breast cancer cases among women. Numerical and structural alterations involving chromosome 8, with loss of short arm (8p) and gain of long arm (8q), are frequently observed in breast cancer cells and tissues. In this study, we show that most of the human breast tumor cell lines examined display an over representation of 8q24, a chromosomal locus RecQL4 is regionally mapped to, and consequently, a markedly elevated level of RecQL4 expression. An increased RecQL4 mRNA level was also observed in a majority of clinical breast tumor samples (38/43) examined. shRNA-mediated RecQL4 suppression in MDA-MB453 breast cancer cells not only significantly inhibit the *in vitro* clonogenic survival and *in vivo* tumorigenicity. Further studies demonstrate that RecQL4 physically interacts with a major survival factor-survivin and its protein level affects survivin expression. Although loss of RecQL4 function due to gene mutations causally linked to occurrence of human RTS with features of premature aging and cancer predisposition, our studies provide the evidence that overexpression of RecQL4 due to gene amplification play a critical role in human breast tumor progression.

## Introduction

Breast cancer is the most leading cause of cancer related deaths among women [1]. Despite advances in endocrine therapy and novel targeted agents, metastatic breast cancer still remains an incurable disease largely because of genetic complexity of this disease, which confers resistance to many treatment modalities [2], [3]. Breast cancer occurs both in sporadic and hereditary forms. Sporadic breast cancer comprises a overwhelming majority of breast tumors, and only 5–10% of cases are hereditary in nature [4]. Complex karyotypic alterations with potential implications for breast cancer initiation, progression and metastasis have been reported in the literature [5], [6]. A recent study employing a gene expression based meta-analysis has identified several chromosomal regions including gains of 1q41–42, 8q24 and 17q as well as loss of 1p31–21, 8p22–21 and 14q24, etc, that may be causal for breast cancer metastasis [5], [6]. In particular, amplification involving long arms of chromosome 8 has been frequently reported in multiple tumors such as osteosarcoma, prostate cancer, pancreatic cancer, cervical cancer, B-cell acute lymphoblastic leukemia and sacrococcygeal teratoma [7]–[12]. These findings suggest that the structural alterations of chromosome 8 may play a causal role in the process of tumor progression. Identification and functional characterization of potential genes responsible for breast tumorigenesis may lead to new therapeutic strategies for effective clinical management of breast cancer.

Human helicases that share extensive homology with *E.coli* RecQ helicase are essential for maintaining genomic stability [13]. Five RecQ helicase members, have been identified in humans. Mutations in three of which including BLM, WRN and RecQL4 have been linked to distinct human disorders with common characteristics of premature aging and cancer predisposition[14]–[16]. While mutational inactivation of WRN and BLM helicases leads to Werner (WS) and Bloom syndromes (BS) respectively, mutations in RecQL4 lead to three autosomal recessive disorders; Rothmund-Thomson syndrome, Baller-Gerold and RAPADILLINO [16], [17]. While the biological functions of BLM and WRN helicases have been fairly well-established, the similar reports about RecQL4 have recently begun to emerge. An authentic role for RecQL4 in the formation of DNA replication initiation complex has been demonstrated [18]. Furthermore, RecQL4 deficiency in mice leads to growth retardation, early death and other symptoms similar to human RTS patients [19]. Recent *in vitro* studies have shown that RecQL4-deficient fibroblasts isolated from RTS patients are extremely sensitive to genotoxic agents [20], [21] and that RecQL4 participates in diverse DNA repair pathways through interaction with multiple DNA repair proteins [22]–[24].

Increased incidence of osteosarcoma has been observed in RecQL4 deficient RTS patients. On the contrary, elevated RecQL4 expression has also been reported in human sporadic osteosarcoma and prostate tumor samples [25], [26]. These findings suggest that RecQL4 can be a double-edged sword whose loss or gain of expression is crucial for tumorigenic events.

Since over representation of RecQL4 harboring locus 8q24 is frequently observed in breast cancer cells, we hypothesized that RecQL4 plays critical roles in breast carcinogenesis. Consistent with over representation of 8q24, a significantly elevated expression of RecQL4 was observed in most of the breast cancer cell lines. An increased RecQL4 mRNA level was also observed in a majority of clinical breast tumor samples examined. Suppression of RecQL4 by shRNA dramatically increased the apoptotic potential of breast cancer cells. Clonogenic cell lines with stable suppression of RecQL4 displayed a markedly reduced tumorigenic potential *in vivo* with an absolute lack of tumor growth in 4 out of 7 nude mice xenografts. Co-immunoprecipitation analysis revealed that RecQL4 physically interacts with a major survival factor survivin through its C-terminal and helicase domains. Further, RecQL4 suppression in breast cancer cells reduced the expression of survivin after oxidative DNA damage. Our study suggests that RecQL4 probably regulates the survival of breast cancer cells through its interaction with survivin, and RecQL4 targeting may prove to be effective strategy for breast cancer treatment.

## Results

### Genomic Locus of RecQL4 is Amplified in Most of Human Breast Tumor Cell Lines

**Figure 1:**
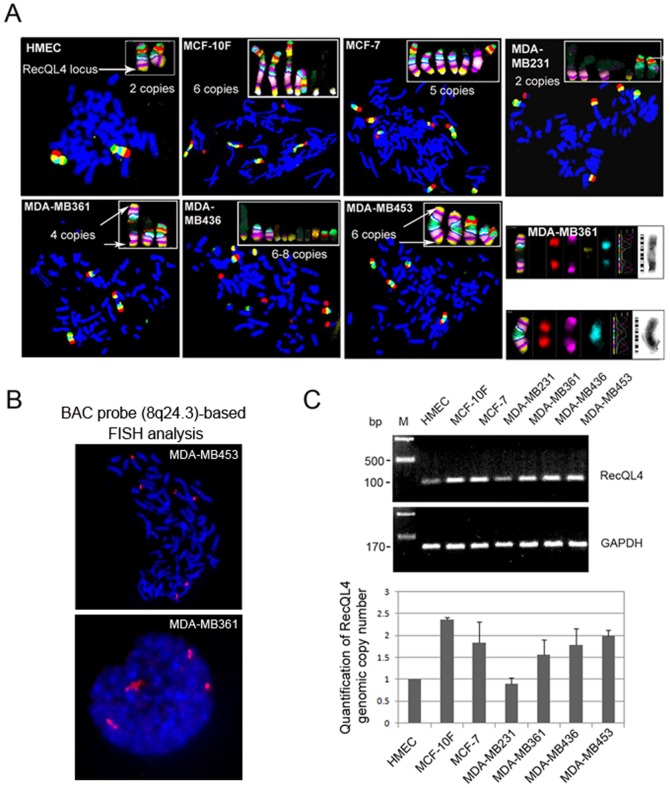
Amplification of RecQL4 genomic locus analyzed by mBAND-FISH and quantitative real time PCR. (A) Analysis of chromosome specific mBAND-FISH in the metaphase spreads of normal primary (HMEC), immortalized (MCF-10F) and tumorigenic breast cancer cell lines. Chromosomes and chromosome regions positive for the probe are shown in the inserts. (B) FISH analysis using spectrum orange labeled BAC (Bacterial Artificial Chromosome) probe proximal to 8q24.3 chromosome locus harboring the RecQL4 gene in MDA-MB453 and MDA-MB361 cells. The BAC probe was purchased from Open Biosystems (RP11–374B7, Huntsville, Alabama, USA). (C) Upper panel: Agarose gel electrophoresis showing the abundance of RecQL4 genomic DNA detected by PCR in breast cancer cell lines relative to normal primary and immortalized breast epithelial cells. GAPDH was used as an internal control; Lower panel: Abundance of RecQL4 genomic DNA detected by quantitative real time PCR in breast cancer cell lines relative to normal primary and immortalized breast epithelial cells. GAPDH was used for normalizing the values of RecQL4.

To determine the alterations involving the loss of chromosome 8p and gain of 8q, FISH using chromosome 8 specific multicolor BAND probe was performed on the metaphase spreads of normal mammary epithelial cells (HMEC) and different breast tumor cell lines. Primary HMEC cells showed two copies of intact chromosome 8 but increased copy numbers (from 4 to 8) with varying 8q lengths (from entire q-arm to distal 1/3 of q) were observed in almost all of the breast cancer cell lines with the exception of MDA-MB231 (Fig. 1A). Interestingly, immortalized non-tumorigenic MCF-10F cell line showed 4 intact copies of chromosome 8 indicating that the over representation of chromosome 8 is most likely associated with immortalization process. Over representation of 8q and under representation of 8p was most evident in MDA-MB453 breast cancer cell line that showed 6 copies of chromosome 8q including a pair of isochromosomes formed by the fusion of two chromosome 8q-arms. Further, FISH analysis using spectrum orange labeled BAC (Bacterial Artificial Chromosome) probe proximal to 8q24.3 chromosome locus harboring the RecQL4 gene was performed in MDA-MB453 and MDA-MB361 cells. Consistent with the mBAND results, the BAC probe revealed 6 hybridization signals in MDA-MB453 cells and 4 hybridization signals in MDA-MB361 cells (Fig. 1B).

To verify whether or not increased copies of chromosome 8q also reflect in the relative enrichment of RecQL4 DNA in breast cancer cell lines, a primer pair corresponding to RecQL4 exon 4 and intron of exons 4–5 was designed, and quantitative PCR using genomic DNA as templates was performed. Consistent with mBAND-FISH data, RecQL4 band intensity and quantitative real-time PCR result in MDA-MB-231 was very similar to primary HMEC cells, whereas an enrichment of RecQL4 DNA was observed in MCF-10F and other four breast tumor cell lines (MCF-7, MDA-MB361, MDA-MB436 and MDA-MB453 cells, Fig. 1C). In corroboration with the copy number analysis by mBAND and quantitative real time PCR analyses, a substantially increased hybridization intensity was observed only in MCF-10F, MCF-7, MDA-MB361, MDA-MB436 and MDA-MB453 cells but not in HMEC normal breast and MDA-MB231 cells (Supplemental Fig. 1).

### RecQL4 Expression is Upregulated in Human Breast Tumor Cell Lines and Clinical Breast Tumor Samples

**Figure 2:**
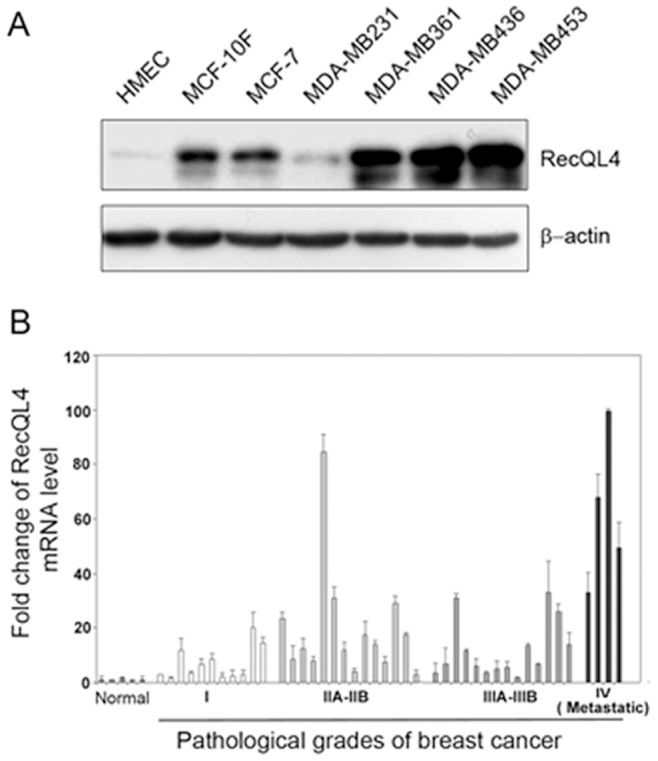
RecQL4 protein or mRNA level in breast tumor cell lines and clinical breast cancer specimens. (A) Western blot detection of RecQL4 protein level in HMEC, MCF-10F and five breast tumor cell lines. β-Actin was used to verify equal loading of proteins. (B) Analysis of RecQL4 expression by quantitative real time PCR in normal breast tissues and breast cancer specimens with different pathological grades. TissueScan breast cancer tissue qPCR array was purchased from Origene. RecQL4 expression detected in normal breast tissues was considered as 1. The data represent mean ± SD from three independent experiments.

Expression of RecQL4 was next monitored at the protein level by western blot using the whole cell lysates of normal breast epithelial cells and cancer cell lines. In corroboration with our earlier results, RecQL4 protein was substantially elevated in all the breast cancer cell lines with the exception of MDA-MB231 relative to primary HMEC cells (Fig. 2A). To verify whether or not RecQL4 expression is elevated in human breast tumor tissue samples, RecQL4 mRNA level was examined using TissueScan Human Breast cancer tissue qPCR array (Origene, Rockville, MD 20850). This array contains cDNA samples of 5 normal breast tissues and 43 breast tumor tissues with different pathological grades. As shown in Figure 2B, 88.4% (38/43) of breast cancer tissues displayed a three-fold higher expression in RecQL4 mRNA than normal tissue samples. Based on the pathological grades, 72.7% (8/11) of stage I, 92.9% (13/14) of stages IIA-IIB, 92.9% (13/14) of stages IIIA-IIIB, and 100% (4/4) of stage IV showed a markedly increased mRNA level of RecQL4. Specifically, RecQL4 mRNA level was increased by an average 62.5-fold in metastatic breast tumors. These data suggest that elevated RecQL4 expression is probably associated with advanced stage of breast carcinogenesis.

### In vitro Clonogenic Survival and in vivo Tumorigenicity of Breast Tumor Cells by shRNA-mediated RecQL4 Suppression

**Figure 3:**
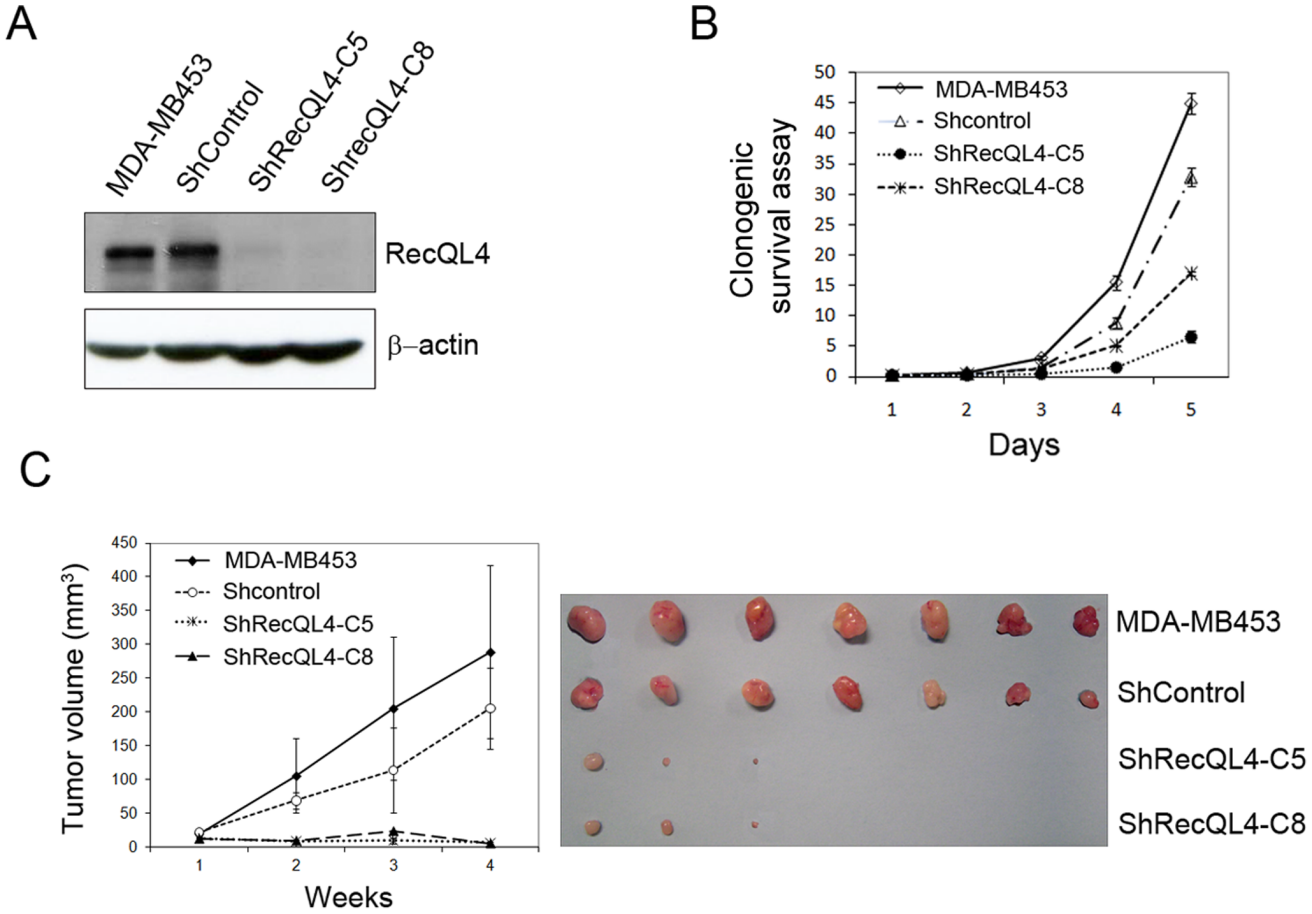
In vitro clonogenic survival and in vivo tumorigenic assays in MDA-MB453 tumor cells after knock-down of RecQL4 expression. (A) Western blot analysis of RecQL4 expression in parental, control shRNA (ShControl) and RecQL4 specific shRNA transduced MDA-MB453 cells (ShRecQL4-C5 & C8). RecQL4 expression was highly reduced in ShRecQL4-C5 and C8 stably selected by puromycin antibiotics. (B) Clonogenic survival assay was performed on ShRecQL4-C5 and C8 cells relative to parental and ShControl cells. Data presented at each time point were the mean value of eight cultures from two independent experiments. Bars indicate mean±SD. (C) Analysis of *in vivo* tumorigenicity of MDA-MB453 cells after RecQL4 silencing. Parental, ShControl and ShRecQL4-C5 & C8 cells were subcutaneously injected into seven immunosuppressed nude mice and tumor growth was monitored for 4 weeks. Tumor growth as a function of time is shown in the left panel. Images of tumors resected from mice are shown in the right panel. Note that 4 of 7 mice injected with either ShRecQL4-C5 or C8 cells did not show any tumor growth. Bars indicate mean±SD.

Elevated level of RecQL4 observed in human breast cancer cells and tissues prompted us to verify whether RecQL4 is crucial for breast cancer cell survival and tumorigenesis. Since MDA-MB453 cells showed a classic over-representation of 8q through isochromosomes formation and underrepresentation of 8p, this cell line was chosen to elucidate the role of RecQL4 in breast carcinogenesis. RecQL4 in MD-MBA453 cells was suppressed by a lentiviral-mediated shRNA approach. Both RecQL4 shRNA and scrambled control shRNA were procured from Santa Cruz Biotechnology. Two clones (designated as C5 and C8) with a substantially decreased level of RecQL4 protein relative to parental and control shRNA-transduced (ShControl) cells were used for further studies (Fig. 3A). Clonogenic survival assay was performed on control and RecQL4 suppressed cell lines. Results presented in Fig. 3B clearly demonstrated that RecQL4 suppression significantly inhibit the clonogenic survival when compared to parental and control ShRNA transduced cell lines. To verify whether or not RecQL4 expression is intimately associated with tumorigenic potential of breast cancer cells, parental MDA-MB453 and control shRNA (ShControl) and RecQL4 specific shRNA transduced cells (ShRecQL4 C5 and C8) were injected subcutaneously into nude mice. The tumor growth was monitored for 4 weeks. Compared to mice injected with parental and control shRNA transduced cells, the tumor size was dramatically reduced in mice injected with RecQL4 suppressed stable cell lines. Interestingly 4 of 7 mice injected with RecQL4 suppressed MD-MBA453 cells failed to show any tumor growth at all (Fig. 3C). These findings clearly illustrate that RecQL4 expression is critical for the tumorigenic potential of breast tumor cells.

### RecQL4 Physically Interacts with Survivin

**Figure 4:**
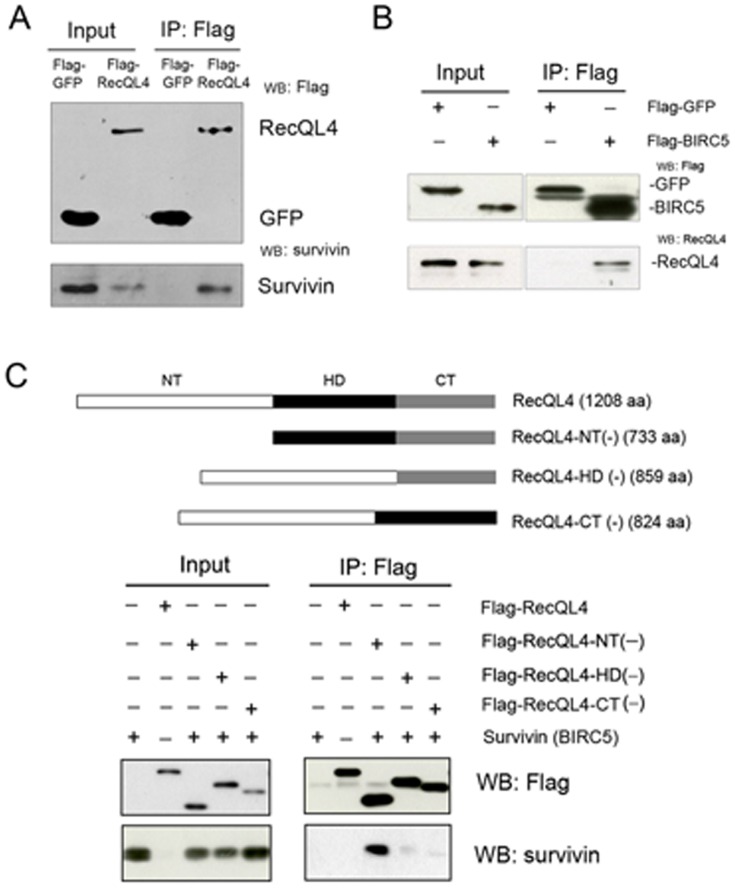
RecQL4 associates with survivin. (A) Endogenous survivin was immunoprecipitated with Flag-RecQL4 recognized by an anti-Flag antibody from cell extracts of 10^6^ U2OS cells, but not with Flag-GFP. The immunoprecipitated proteins were detected with antibody against survivin (Cell Signal). Five percent of the lysate was used for the loading control (Input) and the remaining 95% for co-immunoprecipitation. (B) Endogeneous RecQL4 was immunoprecipitated with Flag-survivin recognized by an anti-Flag antibody from cell extracts of 10^6^ U2OS cells, but not with Flag-GFP. The immunoprecipitated proteins were visualized by Western blot analysis with antibody against RecQL4 (Cell signal). (C) In the upper panel, schematic diagram of RecQL4 deletion constructs used for Co-IP studies is shown. In the lower panel, 293T cells were co-transfected with pRc-CMV2-survivin and one of the Flag-tagged truncated RecQL4 expressing vectors: pFlag-RecQL4-NT(−), pFlag-RecQL4-HD(−) or pFlag-RecQL4-CT(−). Survivin was immunoprecipitated with N-terminal (NT) deleted Flag-RecQL4 protein, not with helicase domain (HD) or C-terminal (CT) deleted Flag-RecQL4 protein.

Our preliminary data has found that level of phospho-histone 3 (p-H3) was substantially decreased in RecQL4-suppressed MDA-MB453 cells. Since survivin has been shown to not only interact physically with histone H3, but also regulate its phosphorylation level [27], [28], we postulated that RecQL4 may modulate p-H3 level through interaction with survivin. To prove this hypothesis, we performed Co-IP (immunoprecipitation) to test the association between RecQL4 and survivin protein using the cell lysates from U2OS cells expressing either full length of Flag-RecQL4 or Flag-survivin. Expression of both genes post transfection was confirmed by Western blotting. When Flag-RecQL4 was precipitated with anti-Flag antibody, a band corresponding to survivin was detected with anti-survivin antibody, demonstrating the physical interaction of RecQL4 with survivin (Fig. 4A). Likewise, endogenous RecQL4 protein was detected in the sample immunoprecipitated with anti-Flag antibody in U2OS cells expressing Flag-survivin (Fig. 4B). To determine which of the RecQL4 functional domains interacts with survivin, various Flag-RecQL4 constructs with deletion of N-terminal (NT, 1–475 aa), helicase domain (HD, 476–824aa) or C-terminal (CT, 825–1208aa) were generated and designated as Flag-RecQL4NT(−), Flag-RecQL4HD(−), and Flag-RecQL4CT(−), respectively (Fig. 4C, upper panel). Each of these truncated RecQL4 constructs was co-expressed with survivin (without Flag tag) in 293 cells and immunoprecipitated with anti-Flag antibody. A similar expression level of truncated RecQL4 was revealed in transfected cells by Western blot analysis. While N-terminal deletion did not affect the interaction between RecQL4 and survivin, deletion of either HD or CT domain of RecQL4 disrupted its interaction with survivin (Fig. 4C, lower panel). These findings indicate that interaction of RecQL4 with survivin is mediated by HD and CT domains.

#### RecQL4 suppression leads to a decreased basal and induced level of survivin

**Figure 5:**
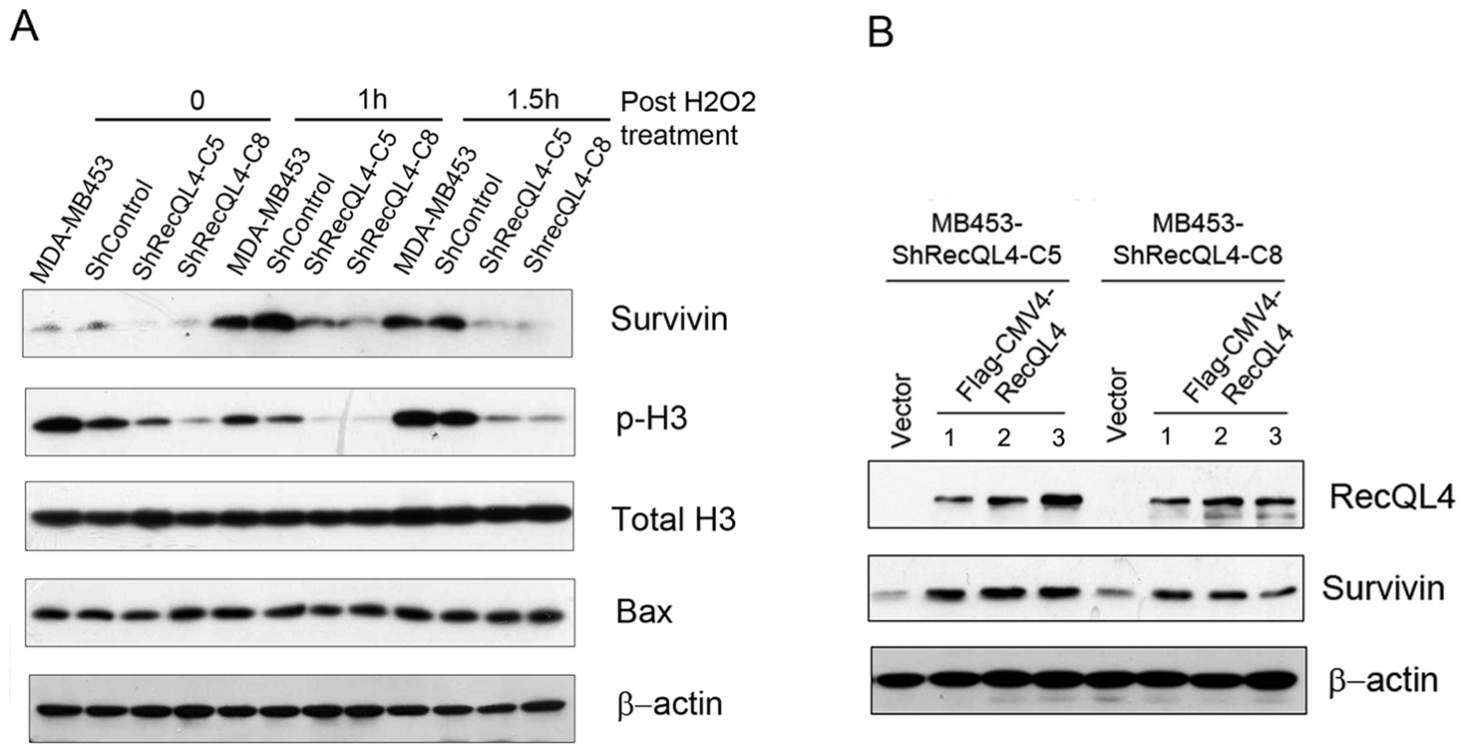
RecQL4 modulates survivin expression. (A)Western blot analysis showed a reduced level of survivin expression and phospho-histone 3 (p-H3) in RecQL4 suppressed cells (ShRecQL4-C5 and C8) at 1 and 1.5 h post 0.25 mM H2O2 treatment. Bax protein did not show any alteration after oxidative DNA damage. β-Actin was used to verify equal loading of proteins. (B) Forced expression of RecQL4 in ShRecQL4-C5 and C8 cells led to an increased level of survivin expression at 48 h post transfection with pFlag-RecQL4 plasmid (lane 1) or plasmids (lane 2 & 3) containing nucleotide substitution at RecQL4 ShRNA-targeted area without changing the amino acid sequence.

Observation of physical interaction between RecQL4 and survivin prompted us to verify whether or not RecQL4 suppression affects survivin expression. In MDA-MB453 cells, RecQL4 suppression reduced the basal level of survivin relative to parental and control shRNA transduced cells (Fig. 5A). The dependence of survivin expression on RecQL4 became more evident when the cells were exposed to hydrogen peroxide (H_2_O_2_). Survivin expression in parental MDA-MB453 cells was greatly induced at 1 h and 1.5 h post H_2_O_2_ treatment, whereas in RecQL4-suppressed cells, survivin induction was substantially inhibited. In addition, phosphorylation of histone H3 (H3ser10), one of the well-known survivin-interacting proteins [29], also showed a much reduced level in RecQL4 suppressed cells (Fig. 5A). We then tested whether or not survivin expression can be induced in RecQL4-silenced MDA-MB453 cells by transiently transfection of either pFlag-CMV4 or pFlag-CMV4-RecQL4 expression vectors. Western blot result revealed an increased level of survivin expression at 48 h post RecQL4 transfection (Fig. 5B).

## Discussion

Cancer metastasis is believed to progress in a multi-step fashion resulting from accumulated gene mutations and amplifications. Among various chromosomal aberrations related to cancer metastasis, chromosomal 8q24 region has been reported to be a frequent site of somatic amplification in a variety of human cancers including breast [6], [30]. Gains of 8q have previously been detected in 50% of the primary breast tumors examined by Comparative genomic hybridization, and have been found to be related to invasive and metastatic potential of breast tumors [31]. By ranking the relation of chromosomal regions to metastasis in a large dataset of more than 1200 breast tumors, RecQL4 at 8q24 has been identified as one of the potential metastasis promoting genes [32]. However, the precise role of RecQL4 in breast tumorigenesis is not entirely known. In this study, we showed Chr. 8q gains and concomitantly, a highly elevated level of RecQL4 protein in breast tumor cell lines. Moreover, a markedly increased mRNA level of RecQL4 was identified in breast tumor tissues with the expression being the highest in metastatic tumor samples. This finding suggests the possibility that RecQL4 may be of use as a prognostic marker for advanced stage of breast cancer.

Unlimited replicative potential and resistance to apoptosis are the hallmarks of human malignancies [33]. In normal cells, accumulation of mutations is prevented through selective elimination of heavily damaged cells by apoptotic death, which is regulated by highly coordinated signal transduction pathways that regulate cell cycle checkpoint and DNA repair activities [34], [35]. Deregulation of these pathways enables the cells to proliferate infinitely through evasion of apoptosis so that cells can tolerate additional gene mutations that are necessary for malignant transformation [36], [37]. Recent studies have shown that RecQL4 plays an authentic role in DNA replisome assembly at DNA replication origin sites [18], [38]. Further, requirement of RecQL4 has been demonstrated for the assembly of Cdc45-MCM2-7-GINS complex in human cells [39], [40]. In the light of these observations, there is a strong possibility that RecQL4 through its elevated expression contributes to infinite replicative potential of breast cancer cells. In support, we demonstrated that RecQL4 suppression led to reduced proliferation potential of MDA-MB453 cells. In addition to proliferation inhibition, RecQL4 suppression also greatly reduced the tumorigenic potential of breast cancer cells *in vivo*. An absolute lack of tumor growth in 4 of 7 mice injected with RecQL4-suppressed breast cancer cells strongly suggests that RecQL4 expression is intrinsically associated with breast cancer cell growth and tumor progression. Association of RecQL4 elevated expression with prostate [26] and breast carcinogenesis indicates the possibility that RecQL4 may be a major replicative helicase for cancer cells.

Genomic instability is thought to play a major role in premature aging and cancer development processes [33]. Three of the human RecQ helicase disorders with mutations in WRN, BLM and RecQL4 helicases exhibit premature aging and cancer susceptibility owing to perturbations in DNA replication, transcription, recombination and repair [41]. RecQL4 deficient RTS displays heterogeneous clinical profiles with common features of premature aging and the early development of cancers, in particular osteosarcomas [42]. In corroboration with pathological symptoms, somatic cells isolated from RTS patients also displayed retarded proliferation and an enhanced sensitivity to agents of oxidative stress and DNA replication blockage [20], [21]. Moreover, RecQL4-deficient cells show genomic instability, including trisomy, aneuploidy and chromosomal rearrangements [43]. These results illustrate the importance of RecQL4 for maintenance of genomic stability in normal cells. In this study, we have demonstrated that shRNA-mediated RecQL4 suppression in breast cancer cells not only inhibits their *in vitro* clonogenic survival and *in vivo* tumorigenicity, but also sensitizes them to apoptotic induction. While loss of RecQL4 in RTS patients leads to osteosarcoma due to compromised DNA metabolic activities [16], gain of expression may favor cancer cell growth and survival through up regulation of DNA replication and repair capabilities. We observed that RecQL4 is most highly expressed in metastatic breast tumors, together with our previous findings in prostate tumors [26] suggests that elevated expression of RecQL4 is potentially significant for metastatic progression of the disease. Thus, RecQL4 may serve as a double-edged sword whose loss or gain of expression is associated with human tumor progression.

Based on our previous report [26], Rb and E2F proteins physically interact with RecQL4 promoter, and Rb hyperphosphorylation correlates with RecQL4 expression in prostate tumor cell lines which is further supported by the data that abolition of Rb hyperphosphorylation by histone deacetylase inhibitor Trichostatin A (TSA) treatment reduced the expression of both E2F1 and RecQL4. Thus, both amplification of RecQL4 genomic locus and the transcriptional mechanism(s) probably contribute to the up-regulation of RecQL4 expression in human cancer cells.

Demonstration of a physical interaction, for the first time in this study, between RecQL4 and survivin indicates the potential importance of RecQL4 in carcinogenic process. Survivin is a unique inhibitor of apoptosis usually expressed in the embryonic lung and fetal organs during the developmental stages but undetectable in most of normal adult tissues [44], [45]. However, selective expression of survivin has been demonstrated in both transformed cells and most human cancers [46], [47]. In addition to apoptosis inhibition, survivin also regulates mitotic spindle checkpoint, and promotes angiogenesis [48]. In this study, we have shown that RecQL4 not only interacts with survivin through HD and CT domains but also seems to regulate survivin expression in the absence and presence of exogenous DNA damage. Although the precise mode of survivin regulation by RecQL4 awaits further studies, our finding suggests that RecQL4 probable protects the breast cancer cells from DNA damage-induced apoptosis through upregulation of survivin.

The multifaceted roles of RecQL4 in diverse DNA metabolic activities have been steadily emerging in recent times [23]. In this study, we have demonstrated that RecQL4 elevated expression confers proliferation advantage and survival to breast cancer cells. The highest level of RecQL4 expression observed in metastatic prostate [26] and breast cancer cells points out a potential prognostic significance of RecQL4 for advanced stages of breast and prostate cancers. Suppression of RecQL4 not only affected the proliferation but also abolished the tumorigenic potential of breast cancer cells. Additionally, RecQL4 through its interaction with survivin seems to be a key factor for determining the fate of breast cancer cells after DNA damage. These novel properties make RecQL4 as an ideal targeting molecule for improving the efficiency of breast cancer treatment. More specifically, RecQL4-survivin pathway provides a potential target for the development of new diagnostic and treatment modalities for breast cancer patients.

## Materials and Methods

### Cell Culture and Reagents

Human breast tumor cell lines (MCF-7, MDA-MB231, MDA-MB361, MD-MBA436 MDA-MB453) were purchased from ATCC (American Type Culture Collection, USA). Cells were cultured in PRMI 1640 medium containing 10% FBS and antibiotics. Primary human mammary epithelial (HMEC) cells were purchased from Invitrogen and cultured in HuMEC serum-free medium (Invitrogen). MCF-10F cells were maintained in DMEM/F12 medium supplemented with 5% horse serum and growth factors (insulin, hydrocortisone and EGF). Antibodies for human RecQL4, survivin, phospho-H3 and total H3 were purchased from Cell Signaling Technology, Boston, MA, USA.

### Plasmid Construction

Full-length human *RecQL4* cDNA was obtained from Genecopoeia and subcloned into NotI/XbaI sites of pFlag-CMV4 vector (Sigma) at NotI/XbaI sites. To generate a series of truncated versions of *RecQL4,* RecQL4 cDNA fragments with 1-824aa (C-terminal deletion) and 476-1208aa (N-terminal deletion) were PCR amplified and cloned into NotI/XbaI sites of pFLAG-CMV4 vector, and were designated as RecQL4-CT(−) and RecQL4NT(−), respectively. For RecQL4 helicase domain (HD)-deleted vector, NT fragment (1-475aa) was first PCR-amplified and cloned into NotI/EcoRI sites of pFlag-CMV4, then CT fragment (825-1208aa) was PCR-amplified and in-frame cloned into EcoRI/XbaI sites to form RecQL4HD(−) vector with deletion of HD domain. All constructs were verified by sequencing and expected sizes of proteins were confirmed by western blot.

The survivin cDNA clone was ordered from Open Biosystems. The survivin cDNA was then PCR amplified and cloned into HindIII-XbaI digested pFlag-CMV4 vector using the following primer set: P1:ACCAAGCTTATGGGAGCTCCGGCG CTG, P2:GCTCTAGATCAGTCCTTATTCTCAATC.

### Plasmid Transfection

The transfection was performed using Lipofectamine reagents (Invitrogen, USA) following the recommended procedure. For transfection in 35 mm dishes, 2 µg of DNA (plasmid) and 8 µl of Plus reagent were diluted in 250 µl serum-free DMEM basal medium, mixed and incubated at room temperature for 15 min. In a separate tube, 12 µl Lipofectamine was diluted in 250 µl serum-free DMEM basal medium, combined with DNA-Plus solution, and incubated at room temperature for additional 15 min. Cells in exponential growth phase with 70–80% confluency in 35 mm dishes were washed once with serum-free medium, and incubated with 2 ml serum-free medium plus Lipofectamine-DNA complexes at 37°C in a humidified incubator for 3h. Then, 2.5 ml medium with 20% FBS was added and the transfection mixture was replaced with complete culture medium after 18–24 h of transfection. MDA-MB453 cells show a high potency for transfection and more than 90% of the cells were observed to be EGFP-positive after transfection with EGFP-expressing vector.

### In situ Fluorescence Hybridization and Chromosome 8 mBAND Analysis

Exponentially growing cells were treated with 0.1 mg/ml Daemecolcine (Sigma) at 37°C for 6–8 hours. Chromosome preparations were made following a standard procedure. For FISH analysis, spectrum orange labeled BAC (Bacterial Artificial Chromosome) probe proximal to 8q24.3 chromosome locus harboring the RecQL4 gene was purchased from Open Biosystems (RP11-374B7, Huntsville, Alabama, USA). For mBAND-FISH analysis, multiple fluorescent DNA probe for chromosome 8 was purchased from Metasystems (Waltham, MA, USA). In brief, slides were washed with 1×PBS twice, incubated with 0.005% pepsin at 37°C for 2 minute, followed by fixation in formaldehyde (3%) for 10 minutes. After washing in 1×PBS, 10–15 µl of the probe was placed on each slide, denatured at 80°C for 3 minutes, and then incubated in a humid environment at 37°C for 2–4 days. After hybridization, the slides were incubated in 1×SSC at 75°C for 5 minutes followed by three washes of 3 min each in 4×SSC/0.05% Tween 20. After rinsing in PBS, the slides were mounted with coverslips using an anti-fade solution containing 200 ng/ml of DAPI. The images were captured and analyzed using Zeiss fluorescence microscope using the Metasystem ISIS software. A total of 10–15 metaphases were analyzed for each cell line.

### Quantitative Real Time RT-PCR

Abundance of RecQL4 genomic locus in normal and breast cancer cells was analyzed by quantitative real-time PCR (Applied Biosystems 7300) using SYBR Green PCR master mix and a pair of primers corresponding to RecQL4 exon 4 and intron of exons 4–5. The primer sequences are P1-TACGGCTCAACATGAAGCAG and P2-CCACATAGGAGGGTCACTGG with expected PCR product size of 117 bp. Quantitative real-time PCR was done in 25 µl reaction volume including100 ng DNA template, 1.5 µl RecQL4 primer set (10pmol/µl) or glyceraldehyde-3-phosphate dehydrogenase (GAPDH, 5pmol/µl) with annealing temperature of 60°C for 30sec. The genomic primer sequences for GAPDH are P1-CGGCTACTAGCGGTTTTACG and P2-AAGAAGATGCGGCTGACTGT with PCR product of 159bp. *GAPDH* level was used as an internal reference gene to normalize the level of DNA template. For real time RT-PCR, the primer pair for RecQL4 is: P1-TCACAGT GAGGTCCCAGATT and P2-CTGACTTCTTGGAAGGCTGA with PCR product of 156 bp. β-actin was used as the internal control for normalization of RecQL4 mRNA. The primer sequences forβ-actin are: P1-CAGCCATGTACGTTGCTAT CCAGG and P2-AGGTCCAGACGCAGGATGGCATG (Origene). Relative RecQL4 genomic copy number or mRNA level was quantified from the comparative threshold cycle (*C*_T_) method as described previously [49].

### Co-IP (coimmunoprecipitation) Assay

For Co-IP, cellular proteins were solubilized in lysis buffer (50 mM Tris-HCl, pH 7.5, 150 mM NaCl, 1% NP-40, 5 mM EDTA, 5 mM EGTA, 20 mM NaF, 0.1 µM PMSF, 0.5 µM benzamidine, 1 µg/µl leupeptin, 1 µg/µl aprotinin, 2 µM microcystin and 0.1 µM NaVO3). Total lysates were incubated with Anti-Flag-M2 magnetic beads (Sigma-Aldrich, St. Louis, MO, USA) at 4°C for 2 h. The beads were washed several times to get rid of aspecific binding of proteins. The beads were finally heated at 80°C in Laemmli buffer and immunoprecipitated proteins were detected by SDS/PAGE and Western blotting using antibodies specific for RecQL4 and survivin.

### shRNA Lentiviral Particles

Human control and RecQL4 specific ShRNA lentiviral particles were procured from Santa Cruz. Cells were transduced with lentiviral particle in the presence of 5 µg/ml polybrene according to the manufacturer’s instructions.

### Clonogenic Survival and Tumorigenicity of RecQL4- Suppressed Breast Cancer Cells

The clonogenic survival assay for parental, control and RecQL4 shRNA transduced (clones C5 and C8) MDA-MB453 cells were performed by plating 1×10^4^ cells in 30 mm dishes. Cell number was determined at 24 h interval for 5 days for each cell line using Coulter counter. Data presented at each time point were the mean value of eight cultures from two independent experiments. Xenograft assay in immunodeficient nude mice was performed essentially as described before [26], [50]. To determine whether *RecQL4* suppression leads to reduced tumorigenicity *in vivo,* 6×10^6^ cells from each of the four cell lines [parental MDA-MB453, empty vector–transfected cells, and two *RecQL4* shRNA–transfected clones (C5 and C8)] were injected subcutaneously into nude mice. Seven mice were used for each treatment. Mice were housed in SPF (specific pathogen free) animal facility, and anesthetized by 1% isoflurane through inhalation before tumor cell injection. Animals were monitored at least two times per week or daily when the mice began showing tumors or decreased activity. In addition, animals were weighted at least three times per week for monitoring their overall health. The tumors were measured using calipers and the tumor size was recorded. The mice will be euthanized if any animals were found unexpectedly to be moribund, rapid body weight loss (over 20% of base line of body weight), hunched posture, lethargy or persistent recumbency, jaundice and/or anemia, or a tumor estimated to be over 10% of the body weight. If the animals display no obvious sign of morbidity, mice will be maintained for up to 4 weeks, then euthanized and processed for tumor analysis. The entire experimental animal protocol and procedure were approved by the Animal Care & Welfare Committee of the Beijing Institute of Genomics (Approved Document Number: 2012A004).

## Supporting Information

Figure S1 **Amplification of RecQL4 genomic locus analyzed by Southern blot analysis. 20** µg of genomic DNA isolated from each of the cell line was digested with 25 U NcoI for 6 h and separated on 0.8% agarose gel by electrophoresis at 40 V for 8 h in TAE buffer. After denaturation and neutralization, the DNA was transferred onto Nylon membrane in 10×SSC buffer and cross-linked with 254 nm UV at 0.125 J/cm2. The membrane was pre-hybridized in 12 mL of Rapid-hybrid buffer (GE, Rapid-hyb Buffer, RPN1635) at 65°C in a rotator for 2 h and then hybridized with 32P-dCTP-labeled probe 1 and probe 2(Takara, Random Primer DNA Labeling Kit, D6045) at 65°C overnight. The membrane was washed with 2×SSC, 1×SSC and 0.1×SSC with 0.1% SDS for 20 min, and then exposed to Kodak film at −80°C for 8 h. The hybridization band density was quantified using the image software (http://rsbweb.nih.gov/ij/).
(TIF)
