# Supplementary figures and images for: RecQL4 Helicase Amplification Is Involved in Human Breast Tumorigenesis
Source: PLoS One. 2013 Jul 22;8(7):e69600. doi: 10.1371/journal.pone.0069600 (PMC3718744; doi:10.1371/journal.pone.0069600)

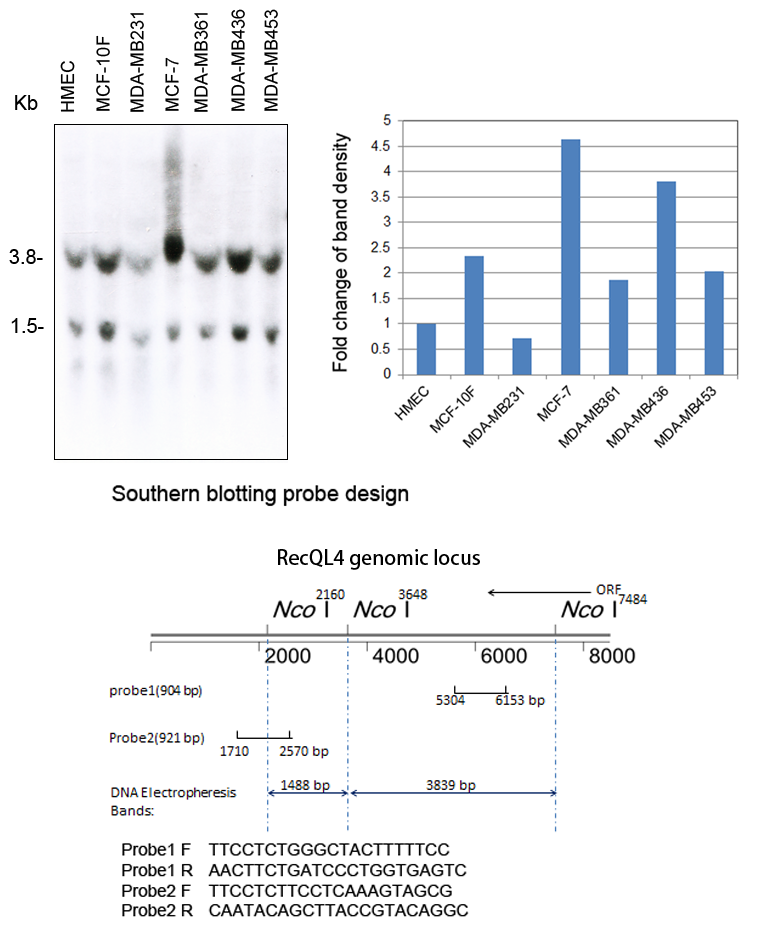

Supplement: Figure S1 — Amplification of RecQL4 genomic locus analyzed by Southern blot analysis. 20 µg of genomic DNA isolated from each of the cell line was digested with 25 U NcoI for 6 h and separated on 0.8% agarose gel by electrophoresis at 40 V for 8 h in TAE buffer. After denaturation and neutralization, the DNA was transferred onto Nylon membrane in 10×SSC buffer and cross-linked with 254 nm UV at 0.125 J/cm2. The membrane was pre-hybridized in 12 mL of Rapid-hybrid buffer (GE, Rapid-hyb Buffer, RPN1635) at 65°C in a rotator for 2 h and then hybridized with 32P-dCTP-labeled probe 1 and probe 2(Takara, Random Primer DNA Labeling Kit, D6045) at 65°C overnight. The membrane was washed with 2×SSC, 1×SSC and 0.1×SSC with 0.1% SDS for 20 min, and then exposed to Kodak film at −80°C for 8 h. The hybridization band density was quantified using the image software (http://rsbweb.nih.gov/ij/). (TIF) [file pone.0069600.s001.tif]
